# Zoning of heavy metal concentrations including Cd, Pb and As in agricultural soils of Aghili plain, Khuzestan province, Iran

**DOI:** 10.1016/j.dib.2017.07.008

**Published:** 2017-07-08

**Authors:** Mehdi Ahmadi, Sahand Jorfi, Amaneh Azarmansuri, Nematollah Jaafarzadeh, Amir Hosein Mahvi, Reza Darvishi Cheshmeh Soltani, Hamideh Akbari, Razegheh Akhbarizadeh

**Affiliations:** a Environmental Technologies Research Center, Ahvaz Jundishapur University of Medical Sciences, Ahvaz, Iran; b Department of Environmental Health Engineering, School of Health, Ahvaz Jundishapur University of Medical Sciences, Ahvaz, Iran; c Department of Environmental Health Engineering, Tehran University of Medical Sciences, Tehran, Iran; d Department of Environmental Health Engineering, School of Health, Arak University of Medical Sciences, Arak, Iran; e Department of Environmental Health Engineering, Health Promotion Research Center and School of Public health, Zahedan University of Medical Sciences, Zahedan, Iran; f Department of Earth Sciences, College of Science, Shiraz University, 71454 Shiraz, Iran

**Keywords:** Soil contamination, Agricultural soil, Cadmium, Arsenic, Lead

## Abstract

Soil is an important component of life cycle affecting agriculture and food crops. Quality of soil resources is defined according to their potential impact on human health by exposure of harmful constituents through the food chain. Heavy metals especially As, Pb and Cd are among the most hazardous elements which could be released to the top soil through different wastewaters, fertilizers, herbicides and etc. In this research Aghili plain in Khuzestan province, Iran was selected as a total of 54 samples were prepared based on a systematic gridding procedure. Selected heavy metals concentrations were analyzed by inductively coupled plasma mass spectrometry (ICP-MS) and then zoning was performed using kriging method. Pollution level was assessed through single factor indices and pollution load index. A separate map dealing with each heavy metal was prepared to present the distribution of heavy metal in Aghili plain. In all samples the heavy metals concentrations were followed the bellow trend: Pb>As>Cd.

Furthermore, based on the PLI, all stations were categorized as moderately to highly polluted sites (1<PLI<4). Due to toxic effects of mentioned heavy metal for human health, furture monitoring, some control measures and remedial actions should be undertaken in the study area.

**Specifications Table**

**Table:** 

Subject area	*Environmental pollution*
More specific subject area	*Soil pollution and monitoring*
Type of data	*Table and Figure*
How data was acquired	*Sampling the designed points of the soil, extraction the samples and analyzing using ICP-MS Spectrometers, Model: SPECTRO ARCOS, Germany*
Data format	*Processed, Raw*
Experimental factors	*Sampling of designed points for determination of soil characteristics and analyzing heavy metal concentration in Aghili plain, Khuzestan province, Iran.*
Experimental features	*Upon sampling and analyzing the obtained data, the map of heavy metal contamination was prepared using kriging method. Descriptive statistics and correlation of variables including Soil characteristics and metal concentrations were performed. Pollution level was assesses using single pollution indices and pollution load index.*
Data source location	*Shushtar city, Khuzestan province, Iran*
Data accessibility	*Data are available in article*

**Value of the data**

• Determination of the concentration of three heavy metals including Pb, Cd and As in agricultural soil was investigated in Aghili plain, Shushtar city, Iran.
• A total of 54 samples were prepared throughout the entire plain according to a systematic girding method.
• Pb concentrations were the highest in all samples compared to As and Cd.
• Zoning of heavy metal concentration was performed and a distribution map was produced for each heavy metal.

## 1 Data

**Fig. 1:**
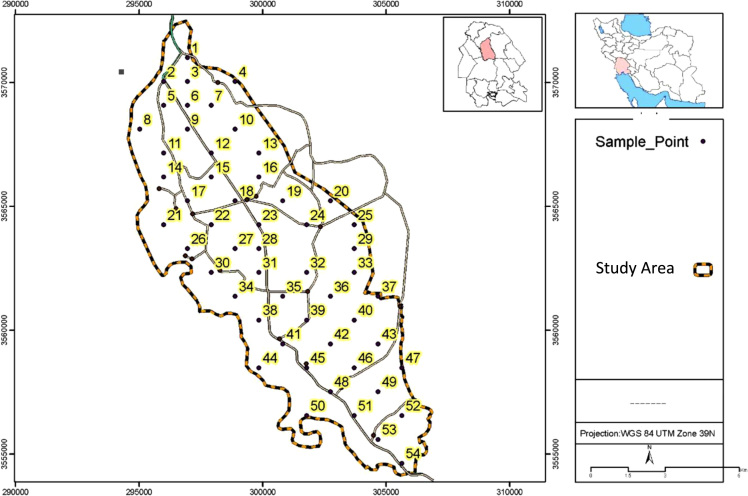
Geographical map of the research area and sampling points.

**Fig. 2:**
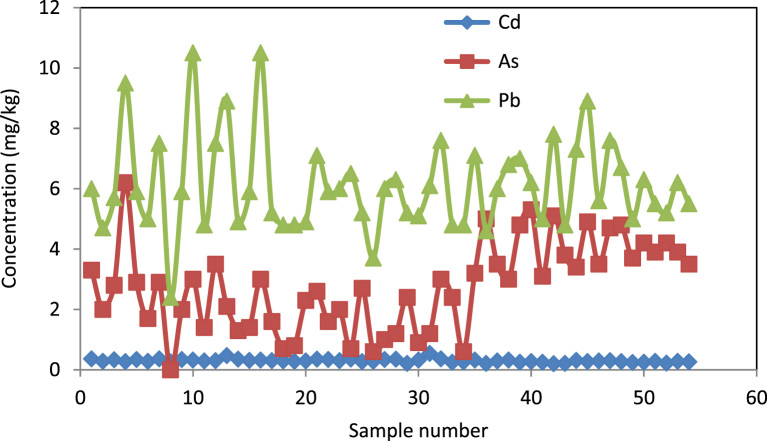
Variations of concentrations of Cd, Pb and As in Aghili plain, Khuzestan province, Iran.

**Fig. 3:**
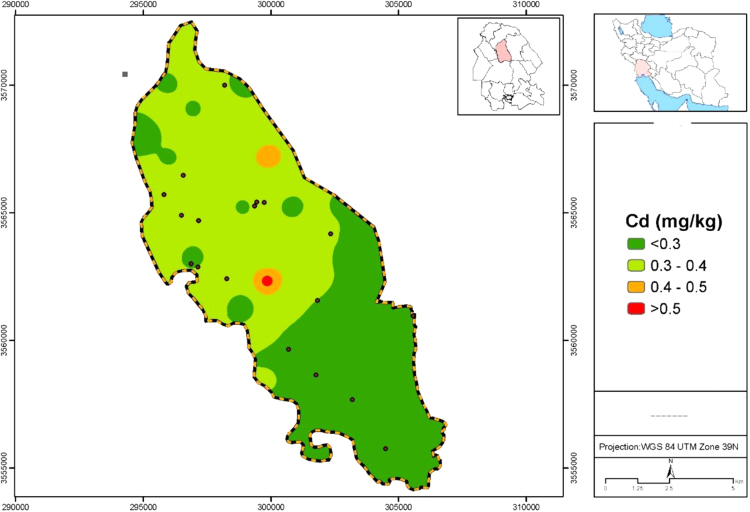
Map dealing with zoning of Cd concentration in surface soil of Aghili plain, Khuzestan province, Iran.

**Fig. 4:**
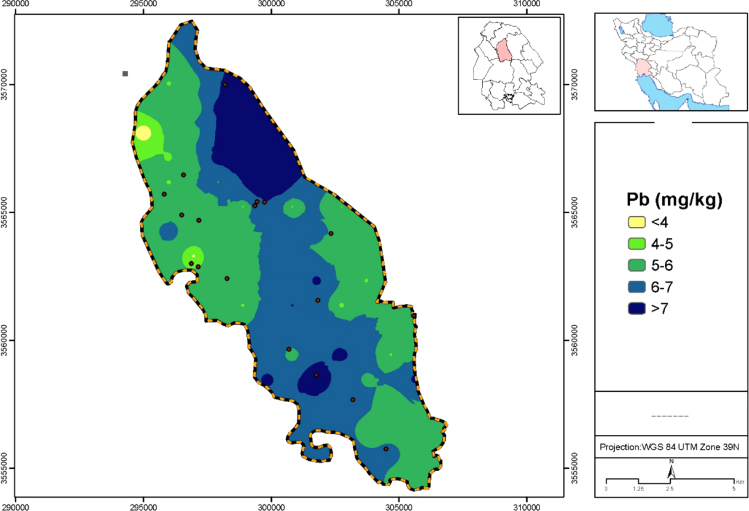
Map dealing with zoning of Pb concentration in surface soil of Aghili plain, Khuzestan province, Iran.

**Fig. 5:**
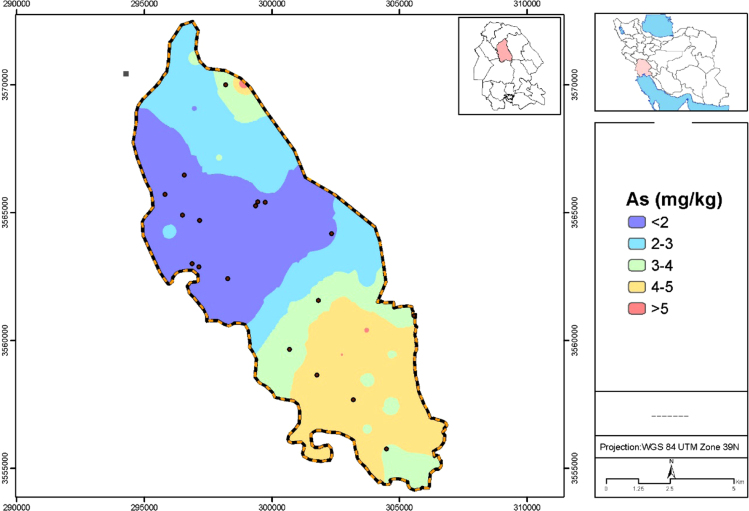
Map dealing with zoning of As concentration in surface soil of Aghili plain, Khuzestan province, Iran.

Data presented here deal with monitoring of selected heavy metals including Cd, Pb and As in Aghili plain, Khuzestan province, Iran. Fig. 1 shows the study area and the sampling points. A summary of characteristics of soil samples are presented in Table 1. Table 2 shows descriptive statistics of results for heavy metal concentrations. The correlation between different variables are presented in Table 3. Results of pollution level assessment are presented in Table 4. Fig. 2 shows the variations of selected heavy metals concentrations including As, Pb and Cd in entire area of research zone. Zonings of Cd, Pb and As in Aghili plain are presented in Fig. 3, Fig. 4, Fig. 5, respectively.

**Table 1:** Soil properties in Aghili plain, Iran.

Parameter	Value
Clay (%)	39.15
Silt (%)	42.56
Sand (%)	18.29
pH	8.12±0.52
TOM (%)	8.24±1.38
EC (ms/cm)	1.69±0.352
Moisture (%)	17.38±2.81

**Table 2:** Descriptive statistical analysis of quantitative monitoring of selected heavy metals in Aghili plain, Khuzestan province, Iran.

Heavy metal	Total samples	Average (mg/kg)	Standard deviation	Maxi (mg/kg)	Min (mg/kg)	Coefficient of variation	Median (mg/kg)	Skewness	Kurtosis
Cd	55	0.299	0.058	0.54	0.2	0.193	0.29	1.535	5.086
As	55	2.81	1.4	6.2	0.4	0.498	2.9	0.175	−0.635
Pb	55	6.124	1.556	10.5	2.4	0.254	5.9	0.886	1.424

**Table 3:** Correlation matrix of studied heavy metals in surface soils of Aghili plain,Iran.

Parameter	As	Pb	Cd	TOM	pH	EC	Moisture
As	1						
Pb	0.446⁎⁎	1					
Cd	−0.385⁎⁎	0.291⁎	1				
TOM	−0.342⁎	−0.135	0.202	1			
pH	−0.158	0.118	0.163	0.032	1		
EC	−0.272⁎	−0.062	0.2	0.175	0.134	1	
Moisture	−0.390⁎⁎	0.091	0.262	0.043	0.1	0.107	1

⁎⁎ Correlation is significant at the 0.01 level (2-tailed).

⁎ Correlation is significant at the 0.05 level (2-tailed).

**Table 4:** Single factor pollution indices (PI) and pollution load index (PLI) for evaluation the level of selected heavy metals pollution in Aghili plain, Iran.

Sample	PI			PLI	Sample	PI			PLI
	As	Cd	Pb			As	Cd	Pb	
1	3	2.76	2.09	2.59	28	1.09	2.69	3.38	2.15
2	1.81	2.15	2.52	2.14	29	2.18	1.61	2.79	2.14
3	2.54	2.53	3.06	2.70	30	0.81	2.46	2.74	1.76
4	5.63	2.07	5.10	3.91	31	1.09	4.15	3.27	2.45
5	2.63	2.61	3.17	2.79	32	2.72	2.76	4.08	3.13
6	1.54	2.15	2.68	2.07	33	2.18	1.92	2.58	2.21
7	2.63	2.76	4.03	3.08	34	0.54	2	2.58	1.41
8	0.09	2	1.29	0.61	35	2.90	2.46	3.81	3.01
9	1.81	2.61	3.17	2.47	36	4.54	1.61	2.47	2.62
10	2.72	2.46	5.64	3.35	37	3.181	2.23	3.22	2.83
11	1.27	2.23	2.58	1.94	38	2.72	2.46	3.65	2.90
12	3.18	2.30	4.03	3.09	39	4.36	1.92	3.76	3.16
13	1.90	3.61	4.78	3.20	40	4.81	2.07	3.33	3.21
14	1.18	2.69	2.63	2.03	41	2.81	1.92	2.68	2.44
15	1.27	2.38	3.17	2.12	42	4.63	1.53	4.19	3.10
16	2.72	2.46	5.64	3.35	43	3.45	1.61	2.58	2.43
17	1.45	2.30	2.79	2.10	44	3.09	2.38	3.92	3.06
18	0.63	2.23	2.58	1.54	45	4.45	2.07	4.78	3.53
19	0.72	2.15	2.58	1.59	46	3.18	2.23	3.01	2.77
20	2.09	2.23	2.63	2.30	47	4.27	2.30	4.08	3.42
21	2.36	2.69	3.81	2.89	48	4.36	2.07	3.60	3.19
22	1.45	2.61	3.17	2.29	49	3.36	1.84	2.68	2.55
23	1.81	2.30	3.22	2.38	50	3.81	1.92	3.38	2.91
24	0.63	2.69	3.49	1.81	51	3.54	2.15	2.95	2.82
25	2.4	2.07	2.79	2.42	52	3.81	1.69	2.79	2.62
26	0.545	2.156	1.987	1.32	53	3.545	2.15	3.333	2.943
27	0.90	2.61	p3.22	1.97	54	3.18	2	2.95	2.65

*Note*: Pollution level based on PI: PI<1 (Non-polluted); 1≤PI<2 (Slight polluted).

2≤PI<3 (Moderately polluted); PI≥3 (Highly polluted) [2], [3].

Note: Pollution level Based on PLI value: PLI=0 background concentration; 0<PLI≤1unpolluted; 1<PLI≤2 moderately to unpolluted; 2<PLI≤3 moderately polluted; 3<PLI≤4 moderately to highly polluted; 4<PLI≤5 highly polluted; PLI>5 very highly polluted [2], [3].

## 2 Experimental design, materials and methods

### 2.1 Sampling procedure

The scope of the sampling area focused on the agricultural area of Aghili plain, Khuzestan province, Iran. Aghili plain has an area of 11,000 ha. A systematic sampling procedure was performed to provide a sampling scheme over the entire plain. The plain was divided into 55 cells of 2 ha in size, within which the topsoil samples (0–20 cm) were collected [1]. A sampling density of one sample per 2 ha was adopted wherever possible in agricultural soils. Each of the soil samples consisted of 5 subsamples collected in a 2 km×2 km grid from the sampling plot with a stainless steel hand auger. For each cell, a total of 1 kg of soil was taken from the mixed samples using a quartile method. The collected soil samples were stored in polyethylene bags for transport and laboratory. The exact location (longitudes and latitudes) of each sample point was measured by GPS instrument.

### 2.2 Statistical analysis

Descriptive statistics including mean, maximum, minimum, median, coefficient of variation (CV), skewness and kurtosis were calculated for samples. The Kolmogorov–Smirnov (K–S) test was applied to check the normality of the variables (significance level was considered at *P*≤0.05). Pearson correlation matrix was also used to identify the relationship between soil variables.

### 2.3 Soil Pollution Assessment

To assess the contamination level of selected heavy metals in Aghili plain, single factor contaminant index (PI) and pollution load index (PLI) were calculated using Eqs. (1) and (2) [2], [3]:

(1) $$\mathrm{PI}\;=\;C_{n}{/B}_{n}$$

(2) $$PLI=\sqrt[{n}]{PI1\times PI2.....PIn}$$

Where C_n_ and B_n_ is the concentration of metal in the soil sample and background, respectively(mg/kg). n is the number of pollutants assessed (i.e., 3) and PIi is the single factor pollution index of each metal. The PLI below 1 implies no pollution whereas PLI greater that 1 shows polluted site. Background concentrations were determined from the mean concentrations of the ghili plain, Iran.

### 2.4 Analytical methods

In order to extract adsorbed Cd, Pb and As in studied soil samples, acid digestion procedure was performed. The collected soil samples were dried for 7 days at 40 °C, sieved through a 2 mm in a plastic sieve and ground to fine powder using agate and a pestle [4]. For the digestion of samples, a representative 2*g* sample was digested with repeated additions (10 mL) of nitric acid (HNO_3_) and hydrogen peroxide (H_2_O_2_) based on USEPA 3050B method. The resultant digestate is reduced in volume while heating and then diluted to a final volume of 100 mL. Particulates in the digestate were removed by centrifugation at 3000 rpm for 10 min [5], [6]. The Limit of Detection (LOD) was evaluated as the ratio of three times of the standard deviation of seven blank readings with respect to the pre-concentration factor as shown in Eq. (3) [7], [8].

(3) LOD=3STD/PF

where, STD is the standard deviation of seven blank readings, and PF is the pre-concentration factor. While the Limit of quantification LOQ was calculated using following equations (4) [7]:

(4) LOQ=3LOD

Physiochemical characteristics of soil samples were also determined. The grain size of soil samples were determined Hydrometer method [9] and the sand, silt and clay content were assessed. Total organic carbon (TOC) content of the soil was determined using the loss on ignition (LOI) method [10]. Soil pH and salinity were measured by mixing soil and distilled water in a 1:2.5 (g:mL) ratio and shaking for 15 min before measuring pH [10].
